# Population Genomics and Morphology Provide Insights into the Conservation and Diversity of *Apis laboriosa*

**DOI:** 10.3390/insects16050546

**Published:** 2025-05-21

**Authors:** Ri Liu, Xuntao Ma, Longfu Zhang, Kang Lai, Changbin Shu, Bin Wang, Mingwang Zhang, Mingxian Yang

**Affiliations:** 1 College of Animal Sciences and Technology, Sichuan Agricultural University, Chengdu 611130, China; 2 Sichuan Provincial Animal Husbandry Master Station, Chengdu 610041, China

**Keywords:** *Apis laboriosa*, whole-genome resequencing, mitochondrial genome, morphological analysis

## Abstract

In recent decades, honeybee populations have declined, dramatically owing to destructive honey harvesting practices and the loss of foraging grounds and nesting sites. Among them, *Apis laboriosa* Smith, 1871 (Hymenoptera, Apidae), an important pollinator species found in the Himalayan region, holds significant economic and ecological value. However, conservation efforts and intraspecific taxonomic studies regarding it have been rather limited, and thus its full geographic range remains elusive. This study is the first to research *A. laboriosa* in Sichuan. Through a systematic study integrating morphological feature analysis and genomic data, the following conclusions are drawn. Whole-genome resequencing data analysis reveals that the Sichuan population forms a new monophyletic group (Bootstraps = 100). In the past ten thousand years, the population sizes of *A. laboriosa* in four different regions of China have been decreasing rapidly. Measures should be taken to protect them across the entire distribution range, especially the populations in Tibet and Sichuan, due to their relatively large genetic differences and low intra-population genetic diversity. Based on the significant difference analysis, the following four wing vein morphological features with extremely significant differences were identified: the width of the right forewing (FB), the cubital index a/b (Ci), the forewing vein angle (E9), and the forewing vein angle (K19). These findings are expected to offer a valuable reference for future *A. laboriosa* conservation endeavors, particularly in protecting populations with a high level of genetic differentiation.

## Simple Summary

The Himalayan giant honeybee (*Apis laboriosa*) is highly adapted to montane environments, constructing nests on cliffs during the breeding season. This nesting behavior makes honey harvesting extremely difficult, resulting in its high market value. Driven by profit, many harvesters use methods such as smoke fumigation and pesticide spraying to expel colonies, leading to significant mortality rates in populations. Reports have shown that *A. laboriosa* populations have declined in certain countries and regions over recent decades. Additionally, the lack of clarity regarding population identity hinders the effective prioritization of conservation targets. Therefore, understanding the genetic differentiation among existing populations is essential for developing conservation plans for this species. Through a systematic integration of morphological feature analysis and genomic data, this study reveals via whole-genome resequencing data analysis that the Sichuan population forms a new monophyletic group (Bootstraps = 100). Over the past 10,000 years, the population sizes of *A. laboriosa* in four different regions of China have rapidly decreased, necessitating conservation measures across their entire distribution range. Significant difference analysis identifies four wing vein morphological characteristics with extremely significant differences. Collectively, this study concludes that the Sichuan population represents a unique geographic population, providing a scientific and theoretical basis for the effective conservation of *A. laboriosa* genetic resources.

## 1. Introduction

Honeybees are important pollinators in ecosystems, and there is a concerning trend of declining wild bee populations globally, there are many threats and challenges to the conservation of honeybees. The main driving factors include anthropogenic factors, as well as climate change [1]. Taxonomic and evolutionary studies of honeybees are crucial for their conservation and for the monitoring of their genetic diversity [2].

The Himalayan giant honeybee, *Apis laboriosa* Smith, 1871, was first described in Yunnan, China, and it is distributed in the Himalayan region. Its unique habitat means that it is not often prioritized for conservation. Since the species has not been domesticated by humans and builds its hives only on cliffs, their honey is not easy to collect and is therefore expensive, costing up to USD 388 per kg in Nepal [3]. Due to the low production and high price of this honey, many collectors drive away bee colonies by spraying pesticides, resulting in the death of many bees. In recent decades, multiple countries and regions have reported declines in bee populations due to habitat destruction and human activities [4,5,6]. In addition, unclear population boundaries pose a major challenge to *A. laboriosa* conservation, making it difficult to effectively prioritize conservation objectives. Therefore, understanding the genetic differentiation of existing populations is necessary to develop effective conservation plans for the species [2].

Originally, *A. laboriosa* was considered to be a subspecies of *Apis dorsata* Fabricius, 1793 [7]. As the taxonomic status of *A. laboriosa* has become widely recognized [8,9], the number of intraspecific studies has increased. In 1983, Kuang et al. [10] concluded that *A. dorsata* and *A. laboriosa* were different species through morphological determination and suggested that the *A. laboriosa* found in Yunnan and Tibet might be different subspecies. Since then, research has focused on the identification of separate species of *A. dorsata* and *A. laboriosa*, and there has been a lack of intraspecific taxonomic studies on *A. laboriosa*. In 2023, Tang et al. [11] revealed the mitochondrial genetic diversity of *A. laboriosa* in Chongqing and Shangri-La and suggested that it may be a subspecies. Subsequently, Cao et al. [12] found evidence that *A. laboriosa* is divided into three populations in China, namely Tibet, western Yunnan, and eastern Yunnan, and revealed their population history and local adaptations through whole-genome resequencing.

In recent years, the distribution range of *A. laboriosa* has been investigated [13]. In 2020, Kitnya et al. [14] summarized the distribution of *A. laboriosa*. The species exhibits a continuous distribution across the Himalayan region, extending eastwards from northern India through Nepal, Sikkim, northern West Bengal, Bhutan, northeastern India, southern Yunnan, and Tibet of China, as well as Myanmar, Laos, and northern Vietnam. Recently, discoveries were made in Chongqing [11] and Thailand [15], and the distribution of the *A. laboriosa* continues to expand. However, there are still gaps and uncertainties in the records from Sichuan, China. In 2024, Otis et al. [16] revisited the distribution of *A. laboriosa* and found that there are only three historical records of *A. laboriosa* in Sichuan Province, and the most recent record is about 40 years ago.

To understand the evolutionary history of *A. laboriosa* in Sichuan Province, this study investigated the distribution of *A. laboriosa* in Ya’an and Ganzi, Sichuan Province. This study aims to systematically investigate the distribution patterns of *A. laboriosa* in Sichuan Province by integrating whole-genome resequencing data, mitochondrial genomic data, and morphological datasets. The findings have provided key distributional information for *A. laboriosa* and basic information for understanding its population structure, genetic diversity, and population history and the development of effective conservation strategies.

## 2. Materials and Methods

### 2.1. Specimen Collection and DNA Sequencing

**Figure 1:**
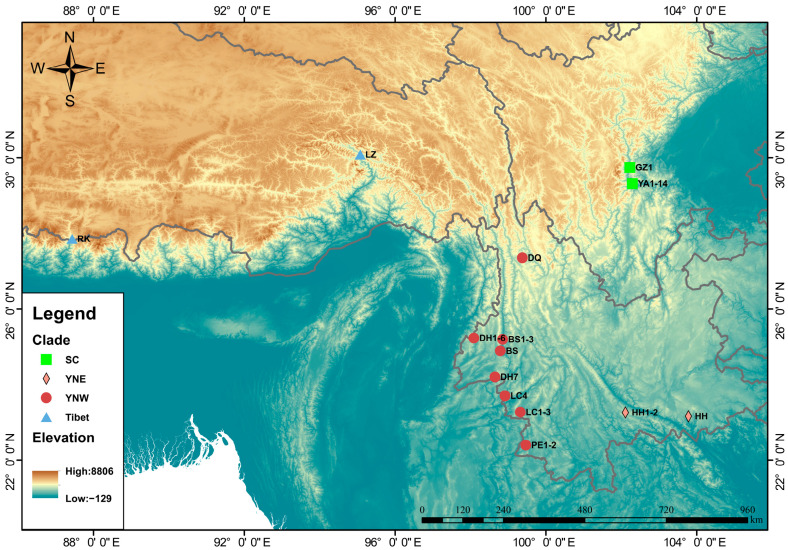
Geographic distribution of 62 Himalayan giant honeybees included in this study. Samples with numbered labels were collected in this study, while unlabeled samples were published samples. Abbreviations: YA = Ya’an (Sichuan Province), GZ = Ganzi (Sichuan Province); HH = Honghe (Yunnan Province), PE = Pu’er (Yunnan Province), BS = Baoshan (Yunnan Province), LC = Lincang (Yunnan Province), DH = Dehong (Yunnan Province), DQ = Diqing (Yunnan Province); RK = Shigatse (Tibet Autonomous Region), LZ = Nyingchi (Tibet Autonomous Region).

Between March 2023 and May 2024, samples were collected from 33 colonies of *A. laboriosa* in the Sichuan and Yunnan provinces (Figure 1). In honeybee colonies, the queen typically mates with multiple drones, resulting in worker bees within a single colony sharing a portion of their genetic material. To mitigate potential biases from intra-colony individuals or genetically related samples in subsequent analyses, one worker bee was selected per colony as a representative, and its thoracic muscle tissue was collected and preserved in anhydrous ethanol [17]. The DNA was subsequently extracted using the Ezup Column Extraction Kit (Sangon Biotech, Shanghai, China), following the manufacturer’s protocol. The extracted DNA was sent to Biomarker Technologies Co., Ltd. (Beijing, China) for sequencing on the Illumina NovaSeq X platform (Illumina, San Diego, CA, USA), which generated paired/linked 150 bp Illumina short reads.

To determine the chromosome locations, the reference genome of *A. laboriosa* (GCA_014066325.1) [18] was compared to the reference genome of *Apis mellifera* Linnaeus, 1758 (GCA_003254395.2) [19] using the Chromosemble program in Satsuma v2.0 [20] to orient the scaffolds to the pseudochromosomes. The new 33 resequencing raw data for *A. laboriosa* in this study are deposited at NCBI under accession number PRJNA1209392. In addition, 29 sequences were downloaded under NCBI accession number PRJNA931733 [12], including samples from Tibet and Yunnan, bringing the total number of sequences ultimately used for subsequent analysis to 62.

### 2.2. Single-Nucleotide Polymorphism Calling

Initially, the raw data for each of 62 *A. laborioda* individuals were filtered out using Seqtk v1.3 [21] by replacing bases with quality scores below 15 with ‘N’ in the resequencing data. Subsequently, the filtered clean reads were mapped to the reference genome using BWA-MEM v0.7.17 [22], with parameters -t 50 -M. Then, SAMtools v1.9 [23] and GATK v4.3 [24] were used to sort BAM files and mark duplicate fragments, respectively. “Mark-Duplicates” in Picard v2.2 (http://broadinstitute.github.io/picard, accessed on 20 August 2024) was used for marking and removing potential PCR duplications. BBMap [25] was used to calculate the average coverage, percent mapped, and percent of reference bases covered for the sequencing data. Finally, single-nucleotide polymorphisms (SNPs) were identified and screened using Sambamba v0.6.5 [26] and BCFtools v1.13 [27] on the tagged duplicates. To avoid including low-quality SNPs, the SNPs were filtered using VCFtools v0.1.16 [28] based on the following parameters: Qual = 20/MinMQ = 30, min-alleles = 2, max-alleles = 2, mac = 3, and max-missing = 0.8.

### 2.3. Population Structure, Principal Component Analysis, and Phylogeny Construction

Kinships among bees were calculated using VCFtools v0.1.16 [28] based on high-quality SNP data, and individuals with kinships less than 0.125 [17] were retained for further analyses. Phylogenetic trees were constructed from high-quality SNPs using IQ-TREE v1.6.12 software [29], and the optimal nucleotide substitution model was TVM + F + R2. To mitigate the effects of linked loci, the dataset was filtered using Plink v1.90. Principal component analysis (PCA) was performed using GCTA v1.92 [30] with the extraction of the top 4 principal components (--pca 4), and population structure analysis was conducted via Admixture v1.3.0 [31] by testing cluster numbers (K) ranging from 1 to 5. Additionally, *F*_ST_ and θπ for different populations were calculated using VCFtools v0.1.16 [28], with 50 Kb sliding windows and a 20 Kb step size. When 0 < *F*_ST_ < 0.05, populations exhibit low differentiation; when 0.05 < *F*_ST_ < 0.15, they show moderate differentiation; when 0.15 < *F*_ST_ < 0.25, populations are highly differentiated; and when *F*_ST_ > 0.25, they are classified as extremely highly differentiated [32].

### 2.4. Population History and Gene Flow

The PSMC [33] and SMC++ [34] methods are widely used and highly reliable for estimating population history, and, thus, they were used to estimate changes in the historical effective population size with a mutation rate of 5.3 × 10^−9^ and a generation time of 1 year [35]. TreeMix v1.13 [36] was used to detect population migration events with the parameters “-m 1–10 -k 500”. Then, *D*-statistic analysis using Dsuite v0.5 r44 software [37] was conducted based on the phylogenetic tree structure with *A. dorsata* as the outgroup.

### 2.5. Mitochondrial Phylogeny

The mitochondrial genome of *A. laboriosa* was assembled using NOVOPlasty v4.3.5 [38] and *COI* gene fragments, and 13 protein-coding genes (PCGs) were obtained based on reference annotation information [39]. In addition, mitochondrial genomic data from Chongqing [11] and Nepal [39,40] were downloaded. After sequence alignment of the 13 PCGs using MAFFT [41], the alignments were concatenated, and a phylogenetic tree was constructed using IQ-TREE [29]. The best-fit substitution model was estimated by ModelFinder [42], and *A. dorsata* [40] was used as the outgroup. Both trees were visualized using iTOL (https://itol.embl.de/, accessed on 1 October 2024), and a haplotype network of the *COI* genes was constructed using PopART v1.7 [43].

### 2.6. Morphological Analyses

Most classical morphological traits (including body size) correlate with climatic variables in local habitats and fail to reflect the true evolutionary history of the organism. In contrast, wing venation characteristics demonstrate environmental independence and strongly support subspecies boundaries inferred from nuclear genomic data [2]. Therefore, in this study, the 16 forewing morphological characters described by Ruttner were investigated [44]. These 16 wing vein morphological characteristics are as follows: right forewing length (FL), right forewing breadth (FB), length of cubital vein a, length of cubital vein b, cubital index a/b (Ci), and forewing vein angles designated as A4, B4, D7, E9, J10, L13, J16, G18, K19, N23, and O26. The mean and standard deviation of the morphometric data for each colony were calculated and tested for normality using SPSS 27. Independent sample *t*-tests were conducted for data that obeyed normal distribution and the Mann–Whitney U Test was conducted for data that did not obey normal distribution.

## 3. Results

### 3.1. Population Structure

**Figure 2:**
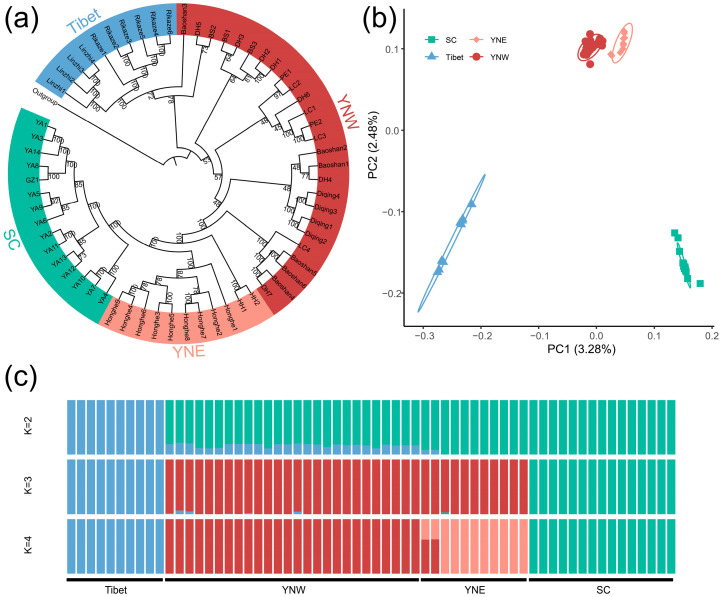
Population genetic structure based on SNPs of the Himalayan giant honeybee. (**a**) Maximum likelihood (ML) phylogenetic tree constructed by IQ-TREE revealing four clusters of Himalayan giant honeybee from different geographical populations: “Tibet”, “YNW” (Western Yunnan), “YNE” (Eastern Yunnan), and “SC” (Sichuan) (ML bootstrap values are indicated at the nodes). (**b**) PCA plot revealing the four distinct genetic clusters. (**c**) A mixture of results with K values ranging from 2 to 4, regarding four ancestral populations (K = 4). Different colors of individuals data points correspond to their cluster identities.

A total of 2,826,775 high-quality SNPs were obtained, and the average sequencing depth was 29.13× (Table S2). The maximum likelihood tree showed clear clustering of the Tibetan populations (bootstrap = 100), though some individuals from the western Yunnan population were nested within it. In addition, the population from Sichuan formed a new monophyletic group with high bootstrap value (Figure 2a). The principal component analysis showed four genetic clusters corresponding to the Tibet, western Yunnan, eastern Yunnan, and Sichuan populations, with principal component (PC) 1 accounting for 3.28% and PC 2 accounting for 2.48%. Therefore, *A. laboriosa* was divided into the Tibetan population, the western Yunnan population, the eastern Yunnan population, and the Sichuan population in China (Figure 2b). For the population genetic structure analysis, five cases of co-ancestral clustering ranging from 1 to 5 were explored, and when K = 2, one cluster contained samples from Tibet and the other cluster consisted of samples from the Yunnan and Sichuan provinces. When K = 3, the samples from Sichuan Province were divided. When K = 4, the samples from Yunnan Province were further divided into eastern and western populations (Figure 2c).

### 3.2. Genetic Differentiation and Genetic Diversity

**Figure 3:**
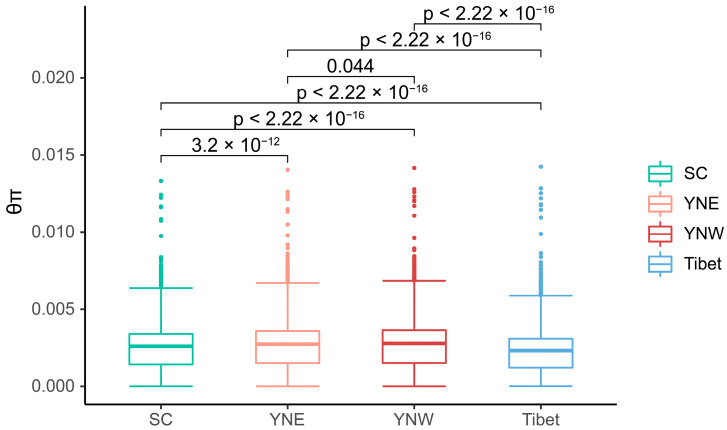
Nucleotide diversity among population samples of *Apis laboriosa* bees computed from genome-wide SNPs using a 50 kb sliding window with a 20 kb step size. The abscissa is four populations (SC, YNE, YNW, Tibet), and the ordinate is the θπ value. In the boxplot of each population, the middle horizontal line represents the median, the box represents the inter-quartile range of the data (25–75%), the upper and lower whiskers are the data range, and the scatter points are outliers.

Pairwise *F*_ST_ values among the four populations ranged from 0.031 to 0.211, with an average of 0.111. According to *F*_ST_ values and the criteria for differentiation levels, the *F*_ST_ values between Tibet and SC (*F*_ST_ = 0.211), as well as between Tibet and YNE (*F*_ST_ = 0.160), fell within the range of 0.15 < *F*_ST_ < 0.25, indicating high differentiation. The *F*_ST_ values between Tibet and YNW (*F*_ST_ = 0.115), between YNE and SC (*F*_ST_ = 0.074), and between YNW and SC (*F*_ST_ = 0.075) were in the interval of 0.05 < *F*_ST_ < 0.15, reflecting moderate differentiation. By contrast, the *F*_ST_ value between YNE and YNW (*F*_ST_ = 0.031) fell within 0 < *F*_ST_ < 0.05, exhibiting low differentiation. The relatively low *F*_ST_ between the Sichuan population and the two Yunnan populations suggests a relationship among these groups. However, the relatively high *F*_ST_ values between the Tibetan population and the other populations suggest that the Tibetan population has significant genetic differentiation. Significant differences in nucleotide diversity were observed among the different populations. Furthermore, the average θπ values of the Tibet, western Yunnan, eastern Yunnan, and Sichuan populations were 0.00222, 0.00265, 0.00261, and 0.00247, respectively (Figure 3), with the lowest value occurring in Tibet and the highest in western Yunnan.

### 3.3. Effective Population Size Analysis

**Figure 4:**
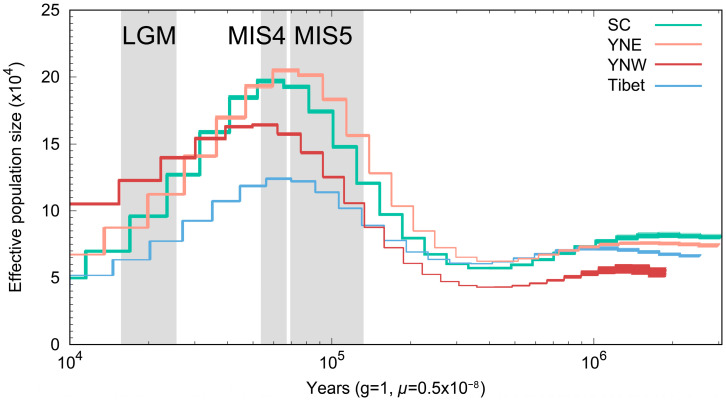
Inference of ancient effective population size history using the PSMC algorithm and stairway plot, with *μ* = 0.5 × 10^−8^ mutations per site per generation and 1 year as generation time.

The population dynamic history was reconstructed for each population using the PSMC model. Four populations had similar changes in effective population size, and all reached a maximum around 70,000 years ago and then leveled off and began to decline (Figure 4). Among them, the populations in Sichuan and eastern Yunnan declined significantly faster than those in western Yunnan.

To confirm these findings, recent population histories were reconstructed using SMC++. Consistent with previous results, the effective population sizes of all four populations exhibited similar trends, with a rapid decline over the last 10,000 years. However, the effective population size of the Sichuan population experienced a large decrease followed by an increase around 20,000 years ago (Figure S1).

### 3.4. Gene Flow

**Figure 5:**
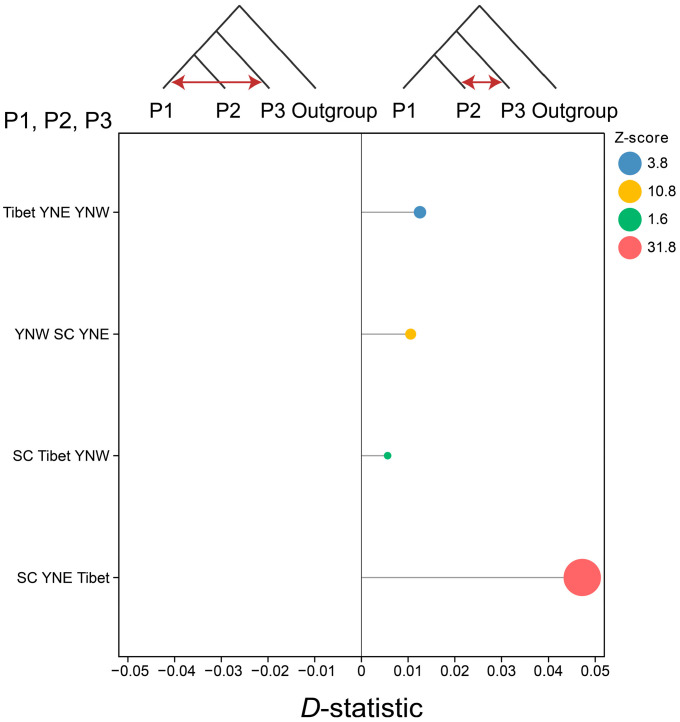
Gene flow between populations estimated using *D*-statistic under the alternative evolutionary models for three populations of *Apis laboriosa* with Giant honeybee *A. dorsata* as the outgroup.

For the *D*-statistic analysis, the horizontal axis represents the *D*-statistic, ranging from −0.05 to 0.05; all calculated *D*-statistics were greater than 0, indicating the presence of gene flow from P3 to P2. The Z-scores for SC YNE Tibet, YNW SC YNE, and Tibet YNE YNW were 31.8, 10.8, and 3.8, respectively, with all exceeding the threshold of 3, indicating significant gene flow (Figure 5). Thus, the results demonstrated gene flow from the Tibetan population to the eastern Yunnan population, from the western Yunnan population to the eastern Yunnan population, and from the eastern Yunnan population to the Sichuan population. Additionally, gene flow from the Tibetan population to the eastern Yunnan population was further supported by TreeMix analysis (Figure S2).

### 3.5. Mitochondrial Phylogeny

We used 13 PCGs from 53 samples and five sequences downloaded from the National Center for Biotechnology Information (NCBI) for phylogenetic tree construction. Unlike the nuclear phylogeny, the maximum likelihood tree based on the mitochondrial genome had two large clades, one of which included the Tibetan population and the other included the Yunnan and Sichuan populations, and the results suggested that the Yunnan and Sichuan populations were related. Except for the Tibetan population, the other populations did not form monophyletic clades. The Sichuan population formed two clades, and one of them included samples from Yunnan (Figure S3). However, the Sichuan population and the two Yunnan populations did not share the same mitochondrial *COI* gene haplotype. Therefore, they can be identified by their *COI* genes. In addition, samples from Chongqing and Nepal clustered into a clade with samples from Linzhi. Based on the haplotype network of the mitochondrial *COI* gene, haplotype sharing only occurred in the western Yunnan population and the eastern Yunnan population (Figure S4). This supports the smaller *F*_ST_ results for these two populations, suggesting less genetic divergence between the two populations.

### 3.6. Morphological Analyses

Independent sample *t*-tests and Mann–Whitney U Tests were conducted on the data (Table S3), and 16 forewing morphometric indicators were measured in 33 colonies of worker bees from Yunnan and Sichuan. The results showed that three morphometrics were significantly different (0.01 < *p* < 0.05) and nine morphometrics were not significantly different (*p* > 0.05). Additionally, right forewing breadth (FB), cubital index a/b (Ci), forewing vein angle (E9), and forewing vein angle (K19) were highly significantly different (*p* < 0.01), and these morphological differences could be used to identify the different populations (Table S4 and Figure S5).

## 4. Discussion

### 4.1. The Status of Sichuan and Tibetan Populations

The *F*_ST_ value between the Tibetan population and the Sichuan population (*F*_ST_ = 0.211) falls within the range of 0.15 < *F*_ST_ < 0.25, indicating high differentiation. Based on the results of phylogenetic tree construction using genome-wide single-nucleotide polymorphisms (SNPs), population genetic structure analysis, principal component analysis (PCA), and mitochondrial *COI* gene haplotype analysis, this study suggests that the Sichuan population represents a distinct geographic population.

In 1983, Kuang et al. [10] suggested that the Tibetan population might be a distinct geographic subspecies based on morphological differences, but this conclusion was not seriously considered due to the small number of samples from Tibet. In this study, the *F*_ST_ value between the Tibetan population and all the other populations (*F*_ST_ = 0.162 ± 0.048) was greater than that among the subspecies of *Apis cerana* Fabricius, 1793 (*F*_ST_ = 0.135 ± 0.06) [2] and was comparable to that in *A. mellifera* (*F*_ST_ = 0.163 ± 0.073) [45]. This suggests that the Tibetan population may have reached the level of subspecific genetic differentiation. However, the subspecies status of the Tibetan population needs to be further confirmed due to the lack of morphological data and our inability to determine the geographic range boundaries of the population.

### 4.2. Changes in Population Size

Changes in effective population size were correlated with historical global climate fluctuations, and during the last major interglacial stage (MIS5; ~70,000–130,000 years ago) [46,47,48] the effective population size increased and reached a maximum. Following MIS5, the effective population size stabilized and began to gradually decline during MIS4. This suggests that past climate change has severely impacted the effective population size of *A. laboriosa*. The observed increase in effective population size during warmer periods suggests that higher global temperatures may have been favorable for *A. laboriosa* populations. Furthermore, the patterns of effective population size changing from the MIS5 phase to MIS4 were similar to those observed in *A. cerana* [49]. After the MIS5 phase, prolonged low temperatures led to a decline in the effective population sizes of all four populations until the end of the Last Glacial Maximum (LGM; ~18,000–27,000 years ago) [50]. Subsequently, the effective population sizes of all four populations either stabilized or recovered, but ultimately declined again until more recent times.

### 4.3. Relationships Between Populations

The *D*-statistic revealed that there was gene flow from the eastern Yunnan population to the Sichuan population, and, according to the population history, the Sichuan population may have migrated from the eastern Yunnan population. Therefore, the Sichuan population may have experienced the founder effect, affecting the rate of population expansion [51]. This may explain the isolation of the Sichuan population and its limited habitat range. In addition, the Sichuan populations are less genetically diverse than the Yunnan populations, which may be because migratory populations are less genetically diverse than their native populations [52].

For the Tibetan and Sichuan populations, the *F*_ST_ was high at 0.211. However, neither the *D*-statistic nor the TreeMix revealed any gene flow between the two populations, which may be because the Hengduan Mountain Range is a barrier to gene flow. The Hengduan Mountain Range is the transition zone between the first and second terraces of China’s topography, with the slope running from northwest to southeast, and undulating terrain that is dominated by high mountains and valleys. The highest peak, Gongga Mountain, is located in the middle of the Hengduan Mountain Range, with an elevation of 7556 m [53]. Therefore, the Hengduan Mountains act as a driver of population divergence [49], limiting gene exchange between the Tibetan and Sichuan populations and resulting in greater population divergence between the two populations.

### 4.4. Mitochondrial Phylogeny

The Yunnan and Sichuan populations did not exhibit mutual monophyly, which may reflect a recent divergence history. However, these differences in phylogenetic structure likely arose due to discrepancies in evolutionary rates between nuclear and mitochondrial genomes. Mitochondrial genes complete genealogical sorting at a faster rate [54,55].

In a previous study, a phylogenetic tree based on mitochondrial genes clustered samples from Chongqing into a clade with those from Nepal [11]. Our results aligned with this finding; however, when data from the Tibetan population were included, samples from Chongqing formed a clade with the Linzhi population. Therefore, we hypothesize that the honeybee colonies in Chongqing likely originated from migratory events originating in the Linzhi region of Tibet.

### 4.5. Conservation Implications

Climate change has induced structural alterations in bee communities and a decline in biodiversity [56]; human factors may also have played an important role [57]. Therefore, measures should be taken to protect the bees’ habitat throughout their range, especially in the Tibetan and Sichuan populations, where the *F*_ST_ is relatively large, and θπ is relatively low.

Wild bees play a critical role in global pollination services, but their populations are undergoing global decline, while managed honeybees (e.g., *A. cerana* and *A. mellifera*) continue to increase in density due to artificial management. High-density managed honeybees may displace wild bees through exploitative competition, threatening biodiversity [58,59]. Therefore, strict preventive ecological assessments must be conducted before introducing beehives in Shimian County, Ya’an City, especially within and around the Liziping National Nature Reserve, to avoid anthropogenic exacerbation of population decline in *A. laboriosa* [60].

The Sichuan population inhabits the area within and around the Liziping National Nature Reserve, which plays a protective role for this population. In 2024, *A. laboriosa* was listed as a key wildlife species for protection in Sichuan Province. In addition, the Liziping National Nature Reserve is rich in native and endemic plant communities. Since *A. laboriosa* is a selective forager [3], the rich plant communities provide more foraging options, which may be one of the reasons why it inhabits this particular area. However, there are still no norms and standards for the collection of honey from this species. Thus, the species is still under great threat, and without the implementation of further measures, some populations are likely to disappear in the coming decades.

For wild bees, addressing intraspecific taxonomic issues is critical to prioritizing conservation efforts as different taxonomic units may face different threats and challenges. This study combined genomic and distributional data to identify the genealogy of obscure endemic populations, assess their population differentiation and genetic diversity, and guide future conservation efforts.

## 5. Conclusions

In this study, the first systematic research on *A. laboriosa* in Sichuan Province was conducted by integrating morphological trait analysis and genomic data, leading to the following specific conclusions. Results from whole-genome resequencing data analysis showed that the Sichuan population formed a new monophyletic group (Bootstraps = 100). Therefore, populations of *A. laboriosa* in China can be divided into four distinct populations: the Tibet population, western Yunnan population, eastern Yunnan population, and Sichuan population. Although fluctuations in effective population size occurred during the evolutionary history of *A. laboriosa*, the sizes of the four regional populations in China have been rapidly decreasing over the past 10,000 years, necessitating measures to protect them across their entire distribution range—especially the Tibet and Sichuan populations, due to their relatively greater genetic differences and relatively lower genetic diversity within populations. Based on analysis of significant differences, substantial genetic differences in partial morphological traits were identified between the Yunnan and Sichuan populations, including four highly significant wing vein morphological traits: right forewing breadth (FB), cubital index a/b (Ci), forewing vein angle (E9), and forewing vein angle (K19). This study first discovered *A. laboriosa* in Garze Prefecture, Sichuan Province, and the finding in Shimian County, Ya’an City confirmed the observation records of *A. laboriosa* in Sichuan Province from approximately 100 years ago. Through integrative analysis of morphology and genomics, this study suggests that the Sichuan population represents a distinct geographic population of *A. laboriosa*. The results of this study provide important insights for the future conservation of *A. laboriosa* and demonstrate the utility of whole-genome resequencing in resolving intraspecific taxonomic challenges for endemic species.

## Data Availability

The resequencing data for *A. laboriosa* are available at NCBI under accession number PRJNA1209392.

## Supplementary Materials

The following supporting information can be downloaded at https://www.mdpi.com/article/10.3390/insects16050546/s1. Table S1. The sample information; Table S2. Statistics of alignment results between 33 resequencing data of *A. laboriosa* and the reference genome; Table S3. Tests of normality for 16 wing vein morphological characters; Table S4. Summary table of morphological data of *A. laboriosa* (mean ± standard deviation); Figure S1. The dynamics of the effective population size of the *A. laboriosa* population were inferred using the SMC++ method; Figure S2. TreeMix is used to infer migration history between populations; Figure S3. Phylogenetic relationships of *A. laboriosa* based on mitochondrial genomes; Figure S4. Mitochondrial *COI* gene haplotype network diagram; Figure S5. Four wing vein morphological features with highly significant differences.
