# Supplementary material for: Population Genomics and Morphology Provide Insights into the Conservation and Diversity of Apis laboriosa
Source: Insects. 2025 May 21;16(5):546. doi: 10.3390/insects16050546 (PMC12112060; doi:10.3390/insects16050546)
Supplement: Supplementary file 1 [file insects-16-00546-s001.zip › insects-3537260-supplementary.pdf]

Supplementary material

Population genomics and morphology provide insights into the conservation and diversity of *Apis laboriosa*

Ri Liu [1] , Xuntao Ma [1] , Longfu Zhang [1] , Kang Lai [2], Changbin Shu [2], Bin Wang [2], Mingwang Zhang [1] and Mingxian Yang [1]

[1] College of Animal Sciences and Technology, Sichuan Agricultural University, Chengdu 611130, Sichuan, China
[2] Sichuan Provincial Animal Husbandry Master Station, Chengdu 610041, China

Table S1. The sample information.

Symbol	Province	Number of colonies	Coordinates	Elevation
YA1-14	Sichuan	14	102°17'33",29°19'00"	1023
GZ1	Sichuan	1	102°13'37",29°44'36"	1751
HH1-2	Yunnan	2	102°06'24",23°15'37"	1780
PE1-2	Yunnan	2	99°28'01",22°23'40"	2219
BS1-3	Yunnan	3	98°51'06",25°11'41"	789
LC1-3	Yunnan	3	99°19'13",23°16'15"	1563
LC4	Yunnan	1	98°54'51",23°41'58"	1460
DH1-6	Yunnan	6	98°05'18",25°13'44"	1846
DH7	Yunnan	1	98°38'52",24°12'03"	1588

Table S2. Statistics of alignment results between 33 resequencing data of *A. laboriosa* and the reference genome.

Sample ID	Percent mapped (%)	Average depth	Coverage (%)
BS1	96.33	42.87	97.43
BS2	84.85	21.11	98.48
BS3	78.88	20.8	98.68
DH1	83.44	33.41	98.85
DH2	78.93	25.96	98.65
DH3	98.21	33.13	98.71
DH4	96.93	25	97.99
DH5	98.02	34.6	98.76
DH6	98.8	27.62	98.25
DH7	96.74	25.68	98.68
GZ1	93.87	42.4	98.77
HH1	96.35	42.6	98.6
HH2	97.2	32.52	98.8
LC1	94.68	41.55	98.96
LC2	83.66	21.81	98.31
LC3	74.45	18.3	98.2
LC4	98.71	30.58	97.63
PE1	56.06	26.09	98.49
PE2	94.09	21.8	95.46
YA1	96.13	48.29	98.8
YA2	96.83	33.74	98.7
YA3	97.53	27.01	98.31
YA4	98.05	21	98.19

YA5	94.11	21.12	98.33
YA6	97.14	23.54	98.57
YA7	98.33	26.56	98.3
YA8	97.2	24.73	98.74
YA9	97.9	24	98.21
YA10	97.59	30.27	98.83
YA11	97	26.07	98.15
YA12	97.19	36.55	98.6
YA13	96.11	20.98	98.33
YA14	98.62	29.49	98.03

Table S3. Tests of normality for 16 wing vein morphological characters.

Morphological indicators	Province	Degree of freedom	Significance
FL	SC	15	0.452
	YN	18	0.152
FB	SC	15	0.975
	YN	18	0.028
Cubital vein a	SC	15	0.619
	YN	18	0.289
Cubital vein b	SC	15	0.682
	YN	18	0.173
a/b（Ci）	SC	15	0.756
	YN	18	0.196
A4	SC	15	0.111
	YN	18	0.704
B4	SC	15	0.745
	YN	18	0.76
D7	SC	15	0.312
	YN	18	0.866
E9	SC	15	0.416
	YN	18	0.112
J10	SC	15	0.987
	YN	18	0.358
L13	SC	15	0.151
	YN	18	0.34
J16	SC	15	0.042
	YN	18	0.054
G18	SC	15	0.266
	YN	18	0.474
K19	SC	15	0.237
	YN	18	0.513
N23	SC	15	0.825
	YN	18	0.686
O26	SC	15	0.971
	YN	18	0.665

Table S4. Summary table of morphological data of *A. laboriosa* (mean ± standard deviation).

Index	Yunnan	Sichuan	*P* - value
FL	13.87±0.31	14.02±0.14	0.068
FB	4.59±0.09	4.65±0.07	0.007*
Cubital vein a	1.2±0.04	1.22±0.03	0.224
Cubital vein b	0.14±0.04	0.17±0.02	0.012

a/b （Ci）	9.77±2.55	7.57±0.81	0.002
A4	37.09±1.18	38.37±1.65	0.014
B4	81.53±1.99	81.89±3.58	0.731
D7	95.77±1.1	95.44±1.55	0.484
E9	18.94±0.62	18.2±0.63	0.002
J10	37.96±1.22	36.98±0.97	0.017
L13	11.54±0.93	11.28±1.22	0.485
J16	98.66±3.44	100.74±1.48	0.219*
G18	89.65±0.83	89±2.8	0.398
K19	66.7±1.1	68.54±2.25	0.009
N23	88.43±2.95	90.28±2.1	0.051
O26	30.84±2.05	30.15±1.65	0.304

Note: * indicates that it is obtained by Mann - Whitney U Test.

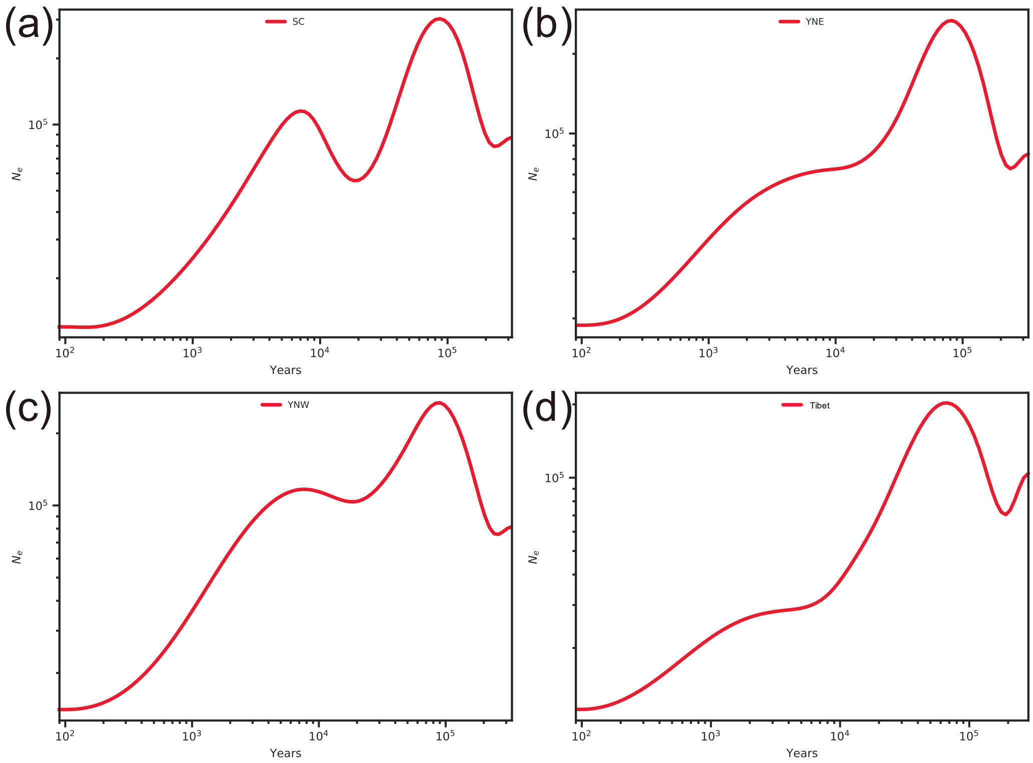

Figure S1. The dynamics of the effective population size of the *A. laboriosa* population were inferred using the SMC++ method.

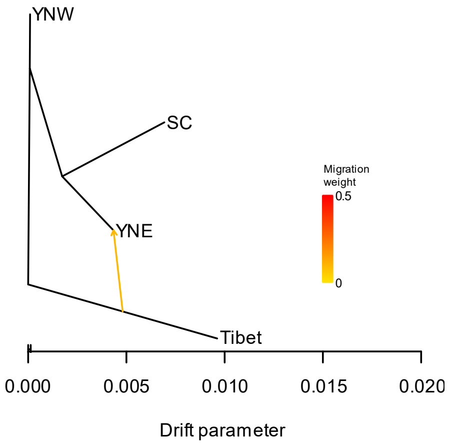

Figure S2. TreeMix is used to infer migration history between populations.

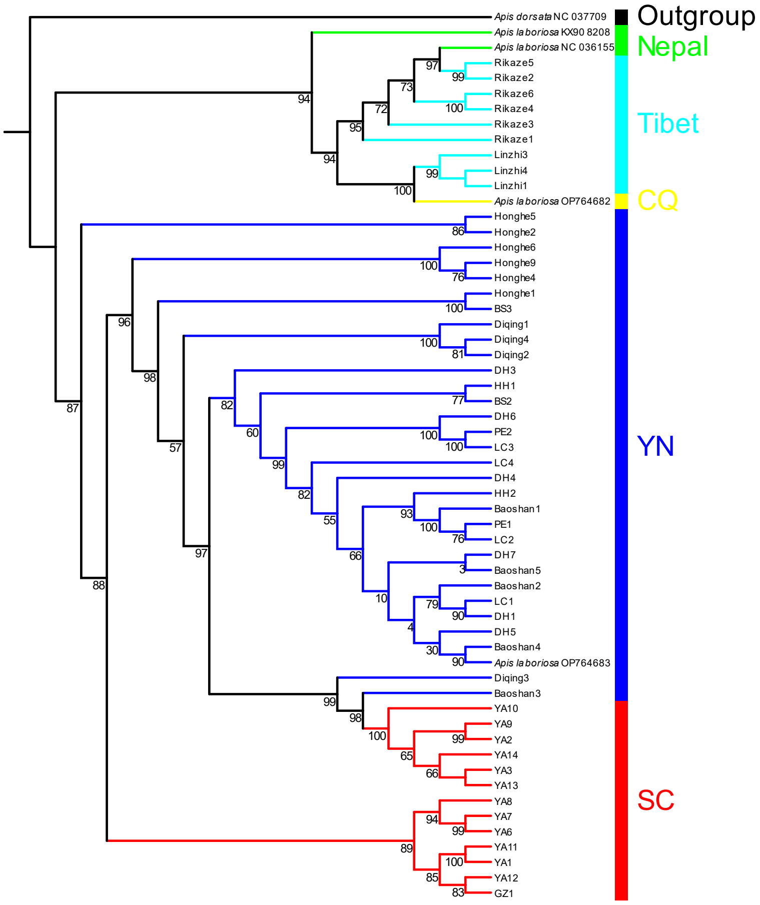

Figure S3. Phylogenetic relationships of *A. laboriosa* based on mitochondrial genomes.

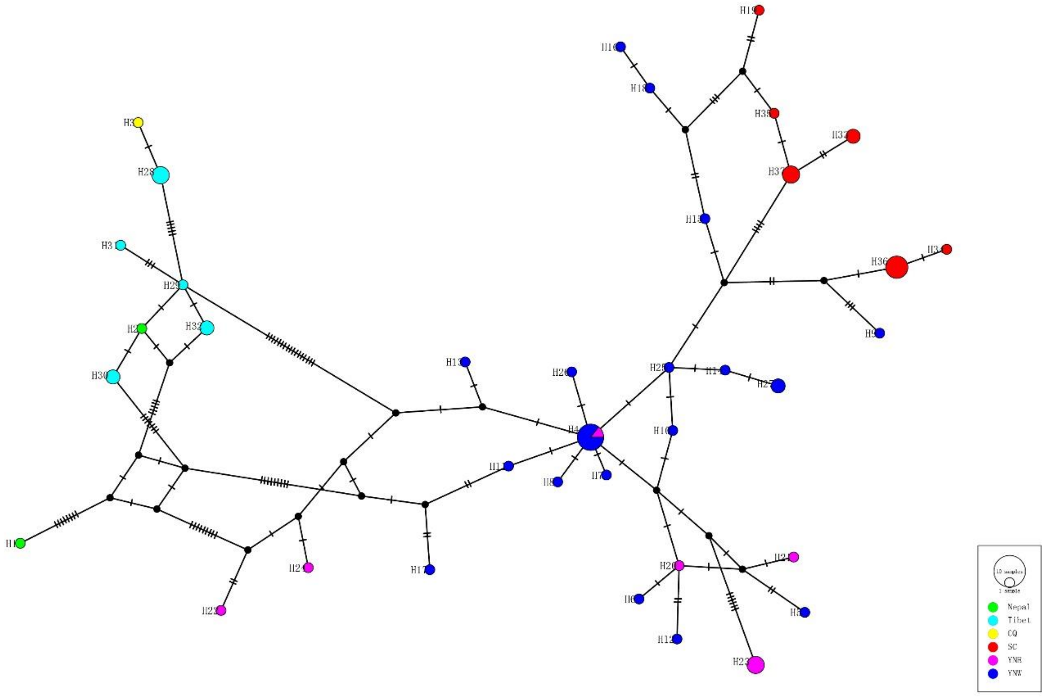

Figure S4. Mitochondrial *COI* gene haplotype network diagram.

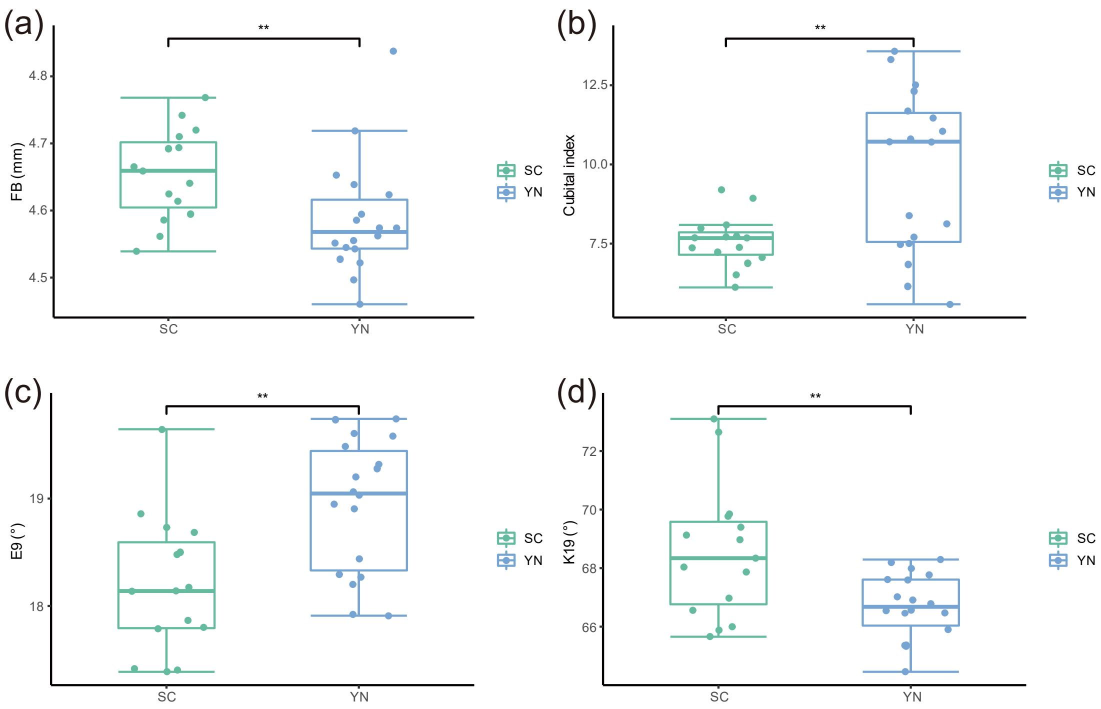

Figure S5. Four wing vein morphological features with highly significant differences.
